# In Vitro Measurement of Genotoxicity of Antimicrobial Dextrin–Polyvinyl Alcohol–Iodine Complex

**DOI:** 10.3390/polym18141771

**Published:** 2026-07-20

**Authors:** Tamara Bukeyeva, Seitzhan Turganbay, Ardak Jumagaziyeva, Zhanar Iskakbayeva, Saltanat Jumabayeva, Anar Seysembekova, Jingcheng Hao, Dana Askarova, Alina Sabdanbekova, Dokturbek Adambekov, Amir Azembayev, Gaukhar Askhatkyzy

**Affiliations:** 1 JSC “Scientific Center for Anti-Infective Drugs”, Almaty 050060, Kazakhstan; bukeyeva_t@mail.ru (T.B.); zhanara_07_74@mail.ru (Z.I.); salta_albann@mail.ru (S.J.); seysembekovaanar@gmail.com (A.S.); askarova.dana08@gmail.com (D.A.); amirakan@mail.ru (A.A.); askhatkyzyg@mail.ru (G.A.); 2 Faculty of Natural Sciences, Kazakh National Women’s Teacher Training University, Almaty 050000, Kazakhstan; 3 Petroleum Engineering Institute “One Belt, One Road”, Kazakh-British Technical University, Almaty 050000, Kazakhstan; 4 Key Laboratory of Colloid and Interface Chemistry, Ministry of Education, Shandong University, Jinan 250100, China; jhao@sdu.edu.cn; 5 Department of Immunology and Virology, Kyrgyz State Medical Academy, Bishkek 720020, Kyrgyzstan; sabdanbekova10@gmail.com (A.S.); d.adambekov@mail.ru (D.A.)

**Keywords:** dextrin, polyvinyl alcohol, iodine, genotoxicity, micronucleus assay, Ames test

## Abstract

Iodine-based antimicrobial materials are widely considered for biomedical applications due to their broad-spectrum antimicrobial activity; however, their potential genotoxicity requires systematic evaluation prior to clinical use. The aim of this study was to assess the genotoxic potential of a dextrin–polyvinyl alcohol–iodine (D/PVA/I-1) complex in accordance with OECD guidelines. Genotoxicity was evaluated in vitro using two complementary assays: the mammalian cell micronucleus test (OECD TG 487) in L5178Y TK^+/−^ cells and the bacterial reverse mutation assay (Ames test, OECD TG 471) using *Salmonella typhimurium* strains TA98, TA100, TA1535, TA1537 and *Escherichia coli* WP2 *uvr*A, both in the presence and absence of metabolic activation (S9). In the micronucleus assay, no statistically significant increase in micronucleus frequency was observed at concentrations ranging from 0.039 to 0.625 mg/mL compared with the negative control. In the Ames test, no increase in revertant colony numbers was detected in any tested bacterial strain at concentrations up to 1250 μg/plate. At higher concentrations, pronounced cytotoxic and bactericidal effects were observed without evidence of mutagenic activity. Overall, the results demonstrate that the D/PVA/I-1 complex does not exhibit genotoxic or mutagenic potential under the tested in vitro conditions, indicating a favorable genetic safety profile and supporting further preclinical evaluation for potential biomedical applications.

## 1. Introduction

Iodine-based antimicrobial materials remain among the most effective approaches for controlling bacterial and fungal infections due to their broad-spectrum antimicrobial activity and the low likelihood of microbial resistance development [1,2]. However, free iodine is characterized by high chemical reactivity, limited stability, and potential cytotoxicity, which restrict its direct use in biomedical applications, including wound dressings and local drug delivery systems [3,4,5]. To overcome these limitations, polymer-based systems for the controlled release of iodine have been actively developed to enhance iodine stabilization and reduce its toxic effects while preserving antimicrobial efficacy [6]. Among biocompatible carrier materials, dextrin and polyvinyl alcohol (PVA) have attracted particular attention owing to their low toxicity, chemical inertness, and ability to form stable hydrogel and composite structures with tunable physicochemical properties. In particular, PVA-based hydrogel systems have been extensively investigated for biomedical applications, including drug delivery systems, wound healing materials, and tissue engineering scaffolds [7,8].

Combined dextrin–polyvinyl alcohol–iodine (D/PVA/I) complexes are considered promising antimicrobial platforms for use in wound dressings, anti-infective surface coatings, and localized delivery systems for bioactive compounds. In our previous study, we demonstrated that the D/PVA/I complex exhibited pronounced antimicrobial activity, controlled iodine release, and an acceptable cytotoxicity profile, supporting its potential as a next-generation antimicrobial material [9]. However, despite these promising biological properties, its genotoxic potential has not yet been investigated, which provided the rationale for the present study. Povidone-iodine formulations have been extensively evaluated for genotoxicity using standard in vitro and in vivo assays, including reverse mutation and micronucleus tests, and no evidence of genotoxic activity has been reported [10,11,12]. However, information regarding the genotoxic potential of novel dextrin–polyvinyl alcohol–iodine complexes remains limited, highlighting the need for the present investigation.

Despite the growing body of evidence supporting the biological activity of iodine-containing polymeric materials, the assessment of their genotoxic potential remains a critical component of preclinical safety evaluation. DNA damage, chromosomal instability, and point mutations are associated with long-term adverse biological consequences, including an increased risk of carcinogenesis [13,14]. Therefore, international regulatory guidelines recommend the use of standardized assays to evaluate the genetic safety of newly developed materials and chemical entities.

According to OECD guidelines, the most informative primary methods for assessing genetic safety are the in vitro mammalian cell micronucleus test (OECD TG 487) and the bacterial reverse mutation assay (Ames test) (OECD TG 471). The combined use of these complementary assays enables a comprehensive evaluation of both chromosomal damage and gene mutations, thereby providing a robust assessment of genotoxic potential [15,16].

Despite extensive investigations of the antimicrobial and physicochemical properties of iodine-containing polymeric materials, information regarding their genetic safety remains limited, particularly for systems based on dextrin and polyvinyl alcohol [13,17]. To date, only a limited number of studies have employed both OECD-recommended assays within a unified experimental framework to comprehensively evaluate the genotoxicity of D/PVA/I systems [15,16]. This knowledge gap restricts the completeness of the toxicological characterization of such materials and may hinder their further biomedical development and application.

This study represents a continuation of our previously published investigations on the antimicrobial activity and cytotoxicity of the D/PVA/I-1 complex. Here, for the first time, a comprehensive assessment of its genetic safety was performed using two complementary OECD-recommended in vitro assays.

The aim of this study was to evaluate the genotoxic and mutagenic potential of the dextrin–polyvinyl alcohol–iodine (D/PVA/I-1) complex using the in vitro mammalian cell micronucleus test (OECD TG 487) and the bacterial reverse mutation assay (Ames test; OECD TG 471), both in the presence and absence of metabolic activation (S9).

## 2. Materials and Methods

### 2.1. Reagents

The test material used in this study was the dextrin–polyvinyl alcohol–iodine (D/PVA/I-1) complex previously developed by our research group [9]. The complex consists of dextrin (molecular weight ~4000 kDa and deacetylation degree of 95%) from Sigma-Aldrich (St. Louis, MO, USA) and poly (vinyl alcohol) from Sigma-Aldrich (molecular weight ~31,000 kDa, St. Louis, MO, USA, ≥99%) as polymeric carriers, with iodine (Labpharm (JSC “Troitsky iodine plant”, Troitsk, Russia, ≥98%) incorporated into the polymer matrix in the presence of potassium iodide. The D/PVA/I-1 formulation contained 4.27 ± 0.15% (*w*/*w*) iodine and was obtained as a dark-brown powder. Detailed information regarding the preparation procedure and physicochemical characterization of the complex has been reported previously [9]. D/PVA/I-1 was selected for the present study because it demonstrated the highest antimicrobial activity together with low cytotoxicity among the developed formulations, making it the most suitable candidate for further preclinical safety evaluation.

All reagents used in this study were of analytical grade, obtained from commercial suppliers, and used without further purification. Mitomycin C and Canada balsam were purchased from AppliChem PanReac/AppliChem (ITW Reagents, Darmstadt, Germany). Cyclophosphamide, rat liver S9 microsomal fraction, Poloxamer 188, glucose-6-phosphate, nicotinamide adenine dinucleotide phosphate (NADP), potassium chloride, Giemsa stain, methyl methanesulfonate, sodium azide, benzo[a]pyrene, 2-aminoanthracene, and 9-fluorenone were obtained from Sigma-Aldrich (St. Louis, MO, USA). RPMI-1640 medium, L-glutamine, trypan blue, and phosphate-buffered saline (PBS) were purchased from Pricella Biotechnology Co., Ltd. (Elabscience, Wuhan, China). Fetal bovine serum and antibiotic–antimycotic solution were obtained from Capricorn Scientific GmbH (Ebsdorfergrund, Germany). Sodium bicarbonate was purchased from Labkhimprom (Almaty, Kazakhstan), glacial acetic acid from Labkhimprom (Moscow, Russia), immersion oil from MiniMed (Bryansk, Russia), and ethanol from Talgar-Spirt (Almaty, Kazakhstan).

### 2.2. Test System and Culture Conditions

The mouse lymphoma cell line L5178Y TK+/− (ATCC CRL-9518, American Type Culture Collection (ATCC), Manassas, VA, USA) was used as the test system for evaluating the clastogenic and/or aneugenic potential of the D/PVA/I-1 complex, in accordance with OECD Guideline TG 487, “In Vitro Mammalian Cell Micronucleus Test” [15]. The cells were cultured in RPMI-1640 medium supplemented with 10% fetal bovine serum (FBS), 2 mM L-glutamine, and 1% antibiotic-antimycotic solution. Cultivation was carried out in a CO_2_ incubator at 37 °C, 5% CO_2_, and 95% relative humidity.

For the bacterial reverse mutation assay (Ames test), standard strains *Salmonella typhimurium* (TA98, TA100, TA1535, TA1537) and *Escherichia coli* WP2 *uvr*A were used in accordance with OECD Guideline TG 471, “Bacterial Reverse Mutation Test” [16]. The characteristics of the test strains used are presented in Table 1.

*Salmonella typhimurium* strains TA1535 and TA1537 and *Escherichia coli* WP2 *uvr*A were obtained from the American Type Culture Collection (ATCC, Manassas, VA, USA), whereas *S. typhimurium* strains TA98 and TA100 were obtained from the Biological Resource Center (NBRC) of the National Institute of Technology and Evaluation (NITE, Tokyo, Japan). All strains were stored at −80 °C until use. Reactivation was performed by inoculating 5 mL of Luria–Bertani (LB) broth, followed by incubation for 18–24 h at 37 ± 1 °C with shaking at 100 rpm until a cell density of approximately 1.5 × 10^8^ cells/mL was reached. Genotypic characterization (his^−^, rfa, *uvr*A/*uvr*B, trp^−^ mutations, presence of R-factor plasmid, and spontaneous reversion frequency) was performed according to the method of Ames and Maron [18,19].

### 2.3. Reference Substances

Mitomycin C (98%) and cyclophosphamide monohydrate (98.9%) were used as positive controls in the micronucleus assay. In the Ames test (bacterial reverse mutation assay), standard mutagens were applied in accordance with OECD TG 471. The characteristics of the compounds used are presented in Table 2.

Positive controls were used in accordance with OECD Guideline TG 487, “In Vitro Mammalian Cell Micronucleus Test,” and OECD Guideline TG 471, “Bacterial Reverse Mutation Test.” Dimethyl sulfoxide (DMSO) and phosphate-buffered saline (PBS) (Sigma-Aldrich, MO, USA) were used as solvents.

### 2.4. Metabolic Activation System

To assess potential genotoxic activity under conditions of metabolic activation, an S9 metabolic activation system (S9 mix) was used. The S9 mix was freshly prepared prior to use and consisted of glucose-6-phosphate (54 mg), NADP (7.5 mg), 150 mM KCl (0.3 mL), sterile distilled water (0.6 mL), and rat liver S9 microsomal fraction (0.6 mL). The metabolic activation system was prepared and applied in accordance with OECD Guideline TG 471, “Bacterial Reverse Mutation Test,” and OECD Guideline TG 487, “In Vitro Mammalian Cell Micronucleus Test.” The final concentration of the S9 fraction in the test system was 10% (*v*/*v*).

### 2.5. Cytotoxicity Assessment and Selection of Test Concentrations

A preliminary cytotoxicity assay was performed to determine the highest analysable concentration of the D/PVA/I-1 complex for the in vitro micronucleus assay. The test concentrations were prepared by serial two-fold (1:1) dilutions in RPMI-1640 medium containing 10% fetal bovine serum, 2 mM L-glutamine, 1% antibiotic-antimycotic solution, and 1% Poloxamer 188. The D/PVA/I-1 complex was readily soluble in RPMI-1640 medium supplemented with 10% fetal bovine serum (FBS), forming a homogeneous solution without visible precipitation under the experimental conditions. The tested concentration range was 2.5–0.039 mg/mL. Exposure to the test substance was carried out both in the presence and absence of metabolic activation (S9), using a 4 h exposure period followed by a 20 h recovery period (Table 3), in accordance with OECD TG 487 [15]. According to OECD TG 487, the highest analysable concentration should ideally induce approximately 55 ± 5% reduction in cell proliferation, as determined by Relative Increase in Cell Count (RICC) or Relative Population Doubling (RPD), while maintaining sufficient cell proliferation for reliable micronucleus scoring. For suspension cell cultures, OECD TG 487 recommends assessing cytotoxicity using either RICC or RPD. Accordingly, RICC was selected for cytotoxicity assessment in the present study.

Relative Increase in Cell Count (RICC) was calculated according to OECD TG 487 using the following equation:

RICC (%) = [(T − T_0_)/(C − C_0_)] × 100

where T_0_ and T represent the initial and final viable cell counts in the treated cultures, respectively, and C_0_ and C represent the corresponding initial and final viable cell counts in the concurrent negative control cultures.

After completion of the 20 h recovery period, the cells were gently resuspended, and a 20 μL aliquot of the cell suspension was mixed with 0.4% trypan blue solution for viable cell counting. Viable cells were counted using a Goryaev counting chamber (MiniMed, Bryansk, Russia) under an inverted microscope (DMI 3000B, Leica Microsystems, Vienna, Austria). The viable cell counts obtained before treatment and after the recovery period were used to calculate RICC according to OECD TG 487. The resulting RICC values were then used to determine the highest analysable concentration for the in vitro micronucleus assay.

Cytotoxicity in the bacterial assay was evaluated based on the reduction in the number of revertant colonies, the decrease in the bacterial background lawn, and the reduction in culture viability [19]. The top agar for *Salmonella* was prepared by dissolving 6.0 g of bacteriological agar (HiMedia Laboratories Pvt. Ltd., Thane, Maharashtra, India), 5.0 g NaCl, 10.5 mg L-histidine, and 12.2 mg biotin in 1000 mL of distilled water, followed by sterilization at 121 °C for 15 min. The top agar for *E. coli* was prepared by dissolving 7.0 g agar, 6.0 g NaCl, and 10.2 mg tryptophan in 1000 mL of distilled water, followed by sterilization at 121 °C for 15 min. To 0.1 mL of an overnight bacterial culture, 0.1 mL of the test substance (5000–310 μg/plate) and 2 mL of top agar were added in the presence or absence of the S9 metabolic activation system. The resulting mixture was poured onto minimal glucose agar plates and incubated at 37 ± 1 °C for 48 h.

### 2.6. In Vitro Micronucleus Assay

L5178Y TK+/− cells were seeded in 96-well plates at a density of 6 × 10^5^ cells/mL. The D/PVA/I-1 complex was tested at concentrations ranging from 0.039 to 0.625 mg/mL. Based on the preliminary cytotoxicity assessment, the concentration range used for micronucleus analysis was selected according to OECD TG 487. Under S9 (−) conditions, concentrations from 0.039 to 0.312 mg/mL were evaluated, whereas under S9 (+) conditions, concentrations from 0.039 to 0.625 mg/mL were included. Mitomycin C (0.1 μg/mL) was used as a positive control in the absence of metabolic activation, whereas cyclophosphamide (10 μg/mL) was used in the presence of metabolic activation. Cells cultured in medium without the D/PVA/I-1 complex served as the negative control. Each test concentration and concurrent negative and positive controls were evaluated using duplicate cultures (*n* = 2) in accordance with OECD Test Guideline 487. Following the 4 h exposure period, the cells were washed twice with fresh culture medium by centrifugation at 130× *g* for 5 min. Fresh culture medium was then added, and the cells were incubated for an additional 20 h at 37 °C in a humidified atmosphere containing 5% CO_2_. Subsequently, cells were subjected to hypotonic treatment, incubated for 10 min at 37 °C, and fixed using a mixture of ice-cold acetic acid and ethanol. The fixed cells were air-dried at room temperature and stained with 3% Giemsa solution for 8 min. Micronucleated cells were scored in 1000 cells in each duplicate culture under a Leica DM2500 microscope (Leica, Vienna, Austria) at 400× magnification. Assay acceptability was evaluated according to the criteria specified in OECD Test Guideline 487, including assessment of cytotoxicity based on the RICC. Statistical analysis was performed using replicate-level data obtained from duplicate cultures. Micronucleus frequencies in each treatment group were compared with the concurrent negative control using Fisher’s exact test. Student’s *t*-test was additionally applied as specified in the predefined study protocol. Statistical significance was defined as *p* < 0.05. Because only duplicate cultures were available for each treatment condition, formal tests of normality were not performed.

### 2.7. Bacterial Reverse Mutation Assay

The bacterial reverse mutation assay (Ames test) was performed using the plate incorporation method in accordance with Ames and Maron [18,19].

Briefly, a single colony of *Salmonella typhimurium* or *Escherichia coli* WP2 *uvr*A was inoculated into 5 mL of Luria–Bertani (LB) broth and incubated overnight at 37 ± 1 °C with shaking at 150 rpm. Subsequently, 0.1 mL of the test substance (D/PVA/I-1) at five concentrations (1250.0, 630.0, 310.0, 40.0, and 10.0 μg/plate), 0.1 mL of an overnight bacterial culture (1 × 10^8^ cells/mL), 0.1 mL of positive or negative control (PBS), and 0.5 mL of the S9 mix or PBS were mixed with 2 mL of top agar and plated onto minimal glucose agar plates. After solidification of the top agar layer, the plates were incubated at 37 ± 1 °C for 48 and 72 h. The number of His^+^ revertant colonies for *S. typhimurium* strains and Trp^+^ revertant colonies for *E. coli* WP2 *uvr*A were counted and compared with the negative control. All concentrations were tested in three independent replicates (triplicates). Colony counting was performed using a colony counter. The results are presented as mean (M) ± standard deviation (SD).

## 3. Results

### 3.1. Cytotoxicity Assessment

A preliminary cytotoxicity assessment of the D/PVA/I-1 complex was performed to determine the concentration range suitable for the in vitro micronucleus assay in accordance with the recommendations of OECD TG 487. The complex was tested at concentrations of 2.5, 1.25, 0.625, 0.312, 0.156, 0.078, and 0.039 mg/mL under both metabolically activated S9 (+) and non-activated S9 (−) conditions.

The calculated RICC values showed a concentration-dependent reduction in cell proliferation with increasing concentrations of the D/PVA/I-1 complex under both S9 (−) and S9 (+) conditions (Table 4).

In the absence of metabolic activation (S9−), concentrations up to 0.312 mg/mL remained analysable, whereas concentrations of 0.625 mg/mL and above produced excessive cytotoxicity and were therefore considered not analysable according to OECD TG 487.

In the presence of metabolic activation (S9+), the highest analysable concentration was 0.625 mg/mL, corresponding to an RICC of 66.7%. Concentrations of 1.25 and 2.5 mg/mL produced excessive cytotoxicity and were therefore considered not analysable.

Although the target cytotoxicity level recommended by OECD TG 487 was not achieved under all experimental conditions, the next higher concentration produced excessive cytotoxicity and was therefore unsuitable for micronucleus analysis. Accordingly, the highest analysable concentration was selected in accordance with OECD TG 487.

Based on the calculated RICC values and the OECD TG 487 recommendation to select the highest analysable concentration without inducing excessive cytotoxicity, the highest analysable concentrations under each experimental condition were selected for the micronucleus assay.

### 3.2. Micronucleus Analysis

**Figure 1:**
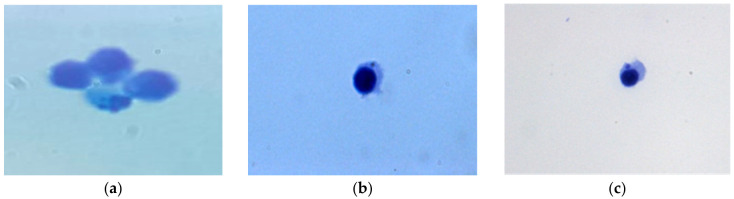
Representative microscopic images of L5178Y cells: (**a**) negative control; (**b**) mitomycin C (positive control, S9 (−)); (**c**) cyclophosphamide (positive control, S9 (+)).

Representative images of L5178Y cells from the negative and positive control groups are presented in Figure 1. Micronuclei were readily observed in cells treated with the reference genotoxic agents mitomycin C and cyclophosphamide, whereas only the background frequency of micronuclei was observed in the negative control.

In the presence of metabolic activation S9 (+), the D/PVA/I-1 complex at concentrations of 0.039–0.625 mg/mL did not induce a statistically significant increase in micronucleus frequency compared with the concurrent negative control at any tested concentration. Similar results were obtained in the absence of metabolic activation S9 (−), where concentrations of 0.039–0.312 mg/mL likewise did not produce a statistically significant increase in micronucleus frequency.

Analysis of the concentration-response relationship revealed no concentration-dependent increase in micronucleus frequency. The frequency of micronucleated cells ranged from 3.5 to 5.5 per 1000 cells (Table 5), which is consistent with the spontaneous background frequency reported for L5178Y cells [13,15,17]. The positive controls (cyclophosphamide and mitomycin C) induced a statistically significant increase in micronucleus frequency compared with the negative control (*p* < 0.05), confirming the validity of the assay. These findings are consistent with previously published studies on povidone–iodine formulations, which also demonstrated no evidence of genotoxicity in standard genotoxicity assays [10,11,12].

Thus, the D/PVA/I-1 complex did not induce a statistically significant increase in micronucleus frequency under either S9 (+) or S9 (−) conditions and showed no evidence of clastogenic effects (chromosome breakage) or aneugenic effects (chromosome loss resulting from mitotic spindle dysfunction) in the in vitro micronucleus assay.

### 3.3. Bacterial Reverse Mutation Assay (Ames Test)

The bacterial reverse mutation assay (Ames test) was performed in accordance with OECD TG 471 to evaluate the mutagenic potential of the D/PVA/I-1 complex [16].

**Figure 2:**
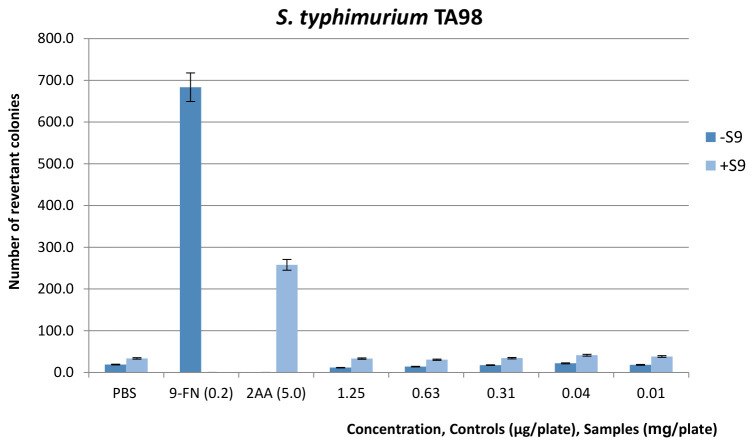
Study results of mutagenic activity of the combined drug based on the D/PVA/I-1 complex with/without metabolic activation on *S. typhimurium* TA98. Values are mean ± SD of three plates. Negative control (NC): PBS (100 μL/plate); positive control (PC): for TA98/S9 (−)-9-FN (concentration 0.2 μg/plate); for TA98/S9 (+)-2-AA (concentration 5.0 μg/plate).

**Figure 3:**
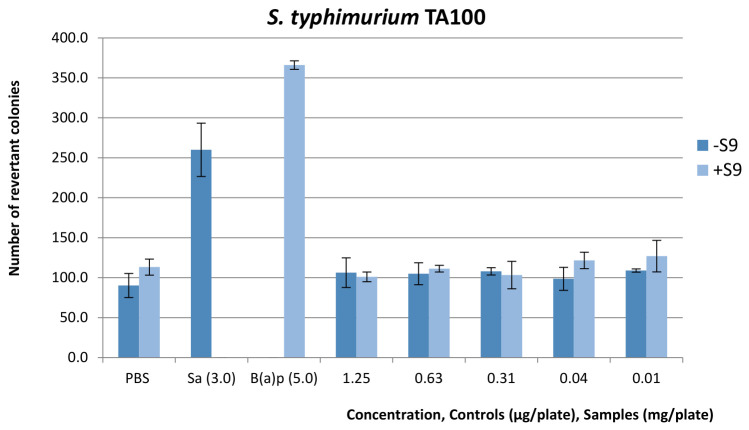
Study results of mutagenic activity of the combined drug based on the D/PVA/I-1 complex with/without metabolic activation on *S. typhimurium* TA100. Values are mean ± SD of three plates. Negative control (NC): PBS (100 μL/plate); positive control (PC): for TA100 without metabolic activation S9 (−)-SA (concentration 3.0 μg/plate); for TA100 with metabolic activation S9 (+)-B(a)p (concentration 5.0 μg/plate).

**Figure 4:**
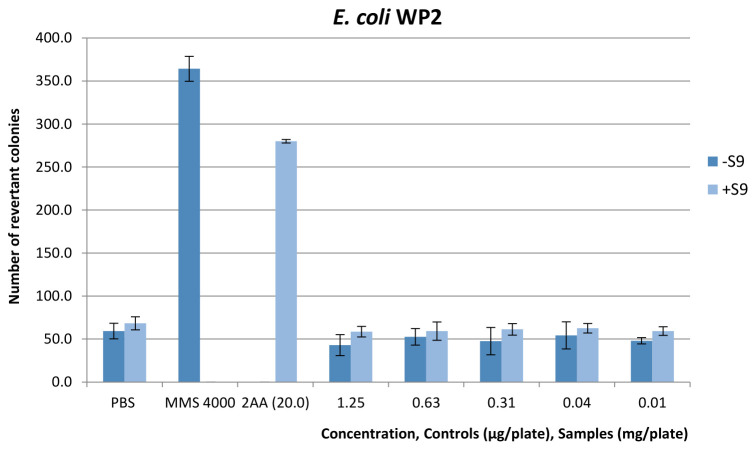
Study results of mutagenic activity of the combined drug based on the D/PVA/I-1 complex with/without metabolic activation on *E. coli* WP2. Values are mean ± SD of three plates. Negative control (NC): PBS (100 μL/plate); positive control (PC): for *E. coli* WP2 without metabolic activation S9 (−)-MMS (concentration 4000.0 μg/plate); for *E. coli* WP2 with metabolic activation S9 (+)-2-AA (concentration 20.0 μg/plate).

**Figure 5:**
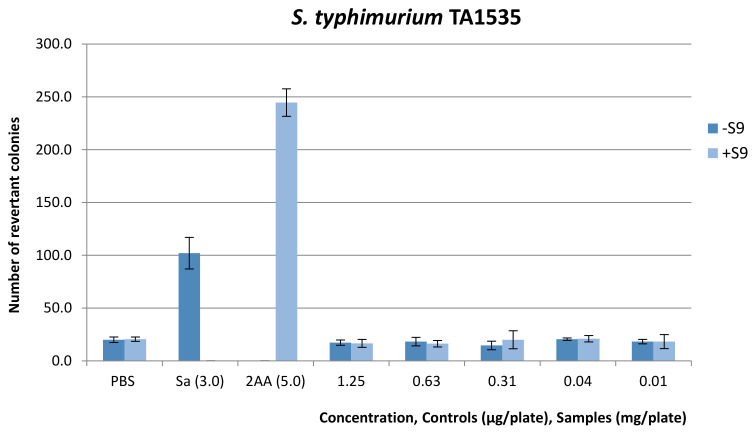
Study results of mutagenic activity of the combined drug based on the D/PVA/I-1 complex with/without metabolic activation on TA1535. Values are mean ± SD of three plates. Negative control (NC): PBS (100 μL/plate); positive control (PC): for TA1535 without metabolic activation S9 (−)-SA (concentration 3.0 μg/plate); for TA1535 with metabolic activation S9 (+)-2AA (concentration 5.0 μg/plate).

**Figure 6:**
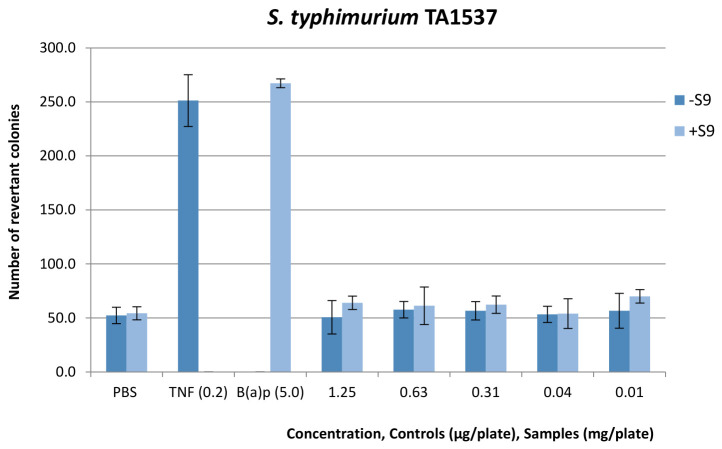
Study results of the mutagenic activity of the combined drug based on the D/PVA/I-1 complex with/without metabolic activation on *S. typhimurium* TA1537. Values are mean ± SD of three plates. Negative control (NC): PBS (100 μL/plate); positive control (PC): for TA1537 without metabolic activation S9 (−)-9-FN (concentration 0.2 μg/plate); for TA1537 with metabolic activation S9 (+)-B(a)p (concentration 5.0 μg/plate).

High concentrations of 5000.0 μg/plate and 2500.0 μg/plate induced pronounced inhibition of bacterial growth in *Salmonella typhimurium* strains TA98 and TA100, as well as *Escherichia coli* WP2, which was manifested by the absence of a bacterial lawn and colony formation. These concentrations were therefore excluded from further analysis (Figure 2, Figure 3, Figure 4, Figure 5 and Figure 6).

The addition of 100 μL of the test substance at concentrations of 5000.0 μg/plate and 2500.0 μg/plate to *S. typhimurium* TA98 cultures completely inhibited bacterial growth, resulting in the absence of colonies on the corresponding plates. At lower concentrations, bacterial growth was observed both in the presence and absence of metabolic activation (S9), while the number of revertant colonies remained comparable to that of the negative control (Figure 2).

Apparently, in Figure 1, application of the D/PVA/I-1 complex on *S. typhimurium* TA98 indicator test strain at concentrations from 0.01 mg/plate to 1.25 mg/plate does not induce the frequency of reversions in comparison with the positive control.

The addition of 100 μL of the test substance at concentrations of 5000.0 μg/plate and 2500.0 μg/plate to *S. typhimurium* TA100 cultures completely prevented bacterial growth, resulting in the absence of colonies on the corresponding plates. At lower concentrations (1250.0, 630.0, 310.0, 40.0, and 10.0 μg/plate), bacterial growth was observed both in the presence and absence of metabolic activation, and the number of colonies remained approximately comparable to the mean colony count in the negative control (Figure 3).

The number of revertants of the *S*. *typhimurium* TA100 test strain induced by the combined drug based on the substance D/PVA/I-1 complex concentration from 0.01 to 1.25 mg/plate statistically did not exceed a background of spontaneous mutations.

The D/PVA/I-1 complex was tested in the *Escherichia coli* WP2 strain at concentrations of 1250.0, 630.0, 310.0, 40.0, and 10.0 μg/plate, both in the absence and presence of the S9 metabolic activation system. The results are presented in Figure 4.

The number of revertants of *E. coli* WP2 test strain induced by application of the D/PVA/I-1 complex in concentration from 0.01 mg/plate to 1.25 mg/plate did not statistically exceed a background of spontaneous mutations.

The D/PVA/I-1 complex was tested in the *Salmonella typhimurium* TA1535 strain at concentrations of 1250.0, 630.0, 310.0, 40.0, and 10.0 μg/plate, both in the absence and presence of the S9 metabolic activation system. The results are presented in Figure 5.

Figure 5 shows that the background of induced mutations when exposed to a combined drug based on the D/PVA/I-1 complex on the *S. typhimurium* TA1535 test strain does not exceed the level of spontaneous mutations, which is also proved by the absence of a significant increase in the level of induced mutations compared with the positive control.

The D/PVA/I-1 complex was tested in the *Salmonella typhimurium* TA1537 strain at concentrations of 1250.0, 630.0, 310.0, 40.0, and 10.0 μg/plate, both in the absence and presence of the S9 metabolic activation system. The results are presented in Figure 6.

Apparently, in Figure 6, the application of a combined drug based on the D/PVA/I-1 complex on the *S. typhimurium* TA1537 indicator test strain in concentration from 0.01 mg/plate to 1.25 mg/plate does not induce the frequency of reversions in comparison with the positive control.

For the evaluation of the toxicity of the D/PVA/I-1 complex, the reduction in the bacterial lawn, changes in the size of microcolonies, and a decrease in the number of revertant colonies were assessed. The *Escherichia coli* WP2 test strain treated with the D/PVA/I-1 complex at concentrations of 1250.0, 630.0, 310.0, 40.0, and 10.0 μg/plate, both in the absence and presence of metabolic activation, showed suppression of the bacterial lawn compared with the control groups. A reduction in bacterial background density and a decrease in the number of revertant colonies were observed in the concentration range of 2500.0–5000.0 μg/plate, indicating pronounced inhibition of bacterial growth.

Based on these results, the maximum analysable concentration was set at 1250 μg/plate. In the main experiment, five concentrations of the D/PVA/I-1 complex were tested: 1250.0, 630.0, 310.0, 40.0, and 10.0 μg/plate (Figure 2, Figure 3, Figure 4, Figure 5 and Figure 6).

According to the data presented in Table 3 and Table 4, treatment of the indicator strain *Salmonella typhimurium* TA98 with the test substance at concentrations ranging from 10.0 to 1250.0 μg/plate did not result in an increase in the number of revertant colonies compared with the negative control. The number of revertants in the *S. typhimurium* TA100 strain also did not exceed the level of spontaneous mutations at any of the tested concentrations. The results demonstrated no statistically significant increase in His^+^ revertants in the treated groups compared with the solvent control (*p* > 0.05). In addition, none of the tested strains showed a dose-dependent response upon exposure to the test substance.

No reduction in the bacterial background and no significant decrease in the number of revertant colonies in *S. typhimurium* TA1535 and TA1537 were observed at any concentration, either in the absence or in the presence of metabolic activation. In the main mutagenicity assay, no increase in the number of revertant colonies was detected following treatment with the D/PVA/I-1 complex.

Thus, the D/PVA/I-1 complex did not induce a statistically significant or biologically relevant increase in revertant colony numbers in any of the tested strains (*S. typhimurium* TA98, TA100, TA1535, TA1537, and *E. coli* WP2 *uvr*A), either in the presence or absence of metabolic activation. The obtained results indicate the absence of detectable mutagenic activity associated with base-pair substitutions and frameshift mutations under the conditions of OECD TG 471.

## 4. Discussion

This study presents a systematic evaluation of the genetic safety of the dextrin-polyvinyl alcohol-iodine (D/PVA/I-1) antimicrobial system using two complementary OECD-validated approaches, enabling the detection of both chromosomal damage and gene mutations within a unified experimental design.

The results of the micronucleus assay in L5178Y TK^+/−^ cells demonstrated no statistically significant increase in micronucleus frequency compared with the negative control, either in the presence or absence of metabolic activation. The obtained values remained within the background level of spontaneous damage, indicating the absence of clastogenic or aneugenic effects across the tested concentration range [13,14,20].

Similar results were obtained in the bacterial reverse mutation assay. In all tested strains of *Salmonella typhimurium* and *Escherichia coli* WP2 *uvr*A, no increase in the number of revertant colonies was observed compared with the spontaneous background level. The absence of a dose-dependent response further supports the lack of induction of both base-pair substitutions and frameshift mutations characteristic of the respective tester strains [19,21,22].

At the same time, pronounced growth inhibition of bacterial and cellular cultures was observed at high concentrations, which is consistent with the known antimicrobial and oxidative properties of iodine, attributable to its ability to interact with functional groups of proteins and lipid components of cellular membranes [1,23,24]. However, the observed effects were predominantly cytostatic and cytotoxic in nature and were not accompanied by an increase in the frequency of genetic damage under in vitro test conditions, indicating the absence of induced mutagenic activity within the investigated concentration range. A similar dissociation between antimicrobial and genotoxic effects of iodine–containing systems has been previously reported for povidone–iodine and related polymer complexes, where strong oxidative activity ensures antimicrobial efficacy without inducing stable DNA damage [1,24,25].

From a mechanistic perspective, the absence of genotoxicity may be attributed to the immobilization of iodine within the polymer matrix of dextrin and polyvinyl alcohol, which potentially reduces its chemical reactivity and limits the release of reactive iodine–containing species. Such a controlled release effect is characteristic of polymeric and hydrogel systems, where the spatial organization of the three-dimensional network restricts the diffusion of active components and thereby decreases their local reactive availability [8,26,27]. Although the precise molecular mechanism remains to be fully elucidated, previous studies have demonstrated that immobilization of iodine within polymer matrices improves its stability, enables controlled iodine release, and reduces the availability of free iodine while preserving antimicrobial activity, which may contribute to reduced nonspecific interactions with biological macromolecules [28,29,30].

A limitation of the present study is that the concentration of iodine released into the culture medium under the conditions of the genotoxicity assays was not directly determined. Since the primary objective of this work was to evaluate the genotoxic potential of the D/PVA/I-1 complex using OECD-recommended assays, quantitative analysis of iodine release kinetics was beyond the scope of the present study. Nevertheless, quantitative determination of iodine release under the experimental conditions used in the present study would provide additional mechanistic insight into the relationship between iodine release kinetics and the observed biological responses and should be addressed in future investigations.

In particular, povidone–iodine has been reported to exhibit no genotoxic potential in standard in vitro and in vivo test systems, including reverse mutation, chromosomal aberration, and micronucleus assays [10,11,12]. Although the available literature generally indicates a favorable genetic safety profile of iodine–containing compounds, incorporation of iodine into a polymeric matrix may alter its release kinetics, bioavailability, and local biological interactions through matrix diffusion effects and interactions within the three-dimensional polymer network. Therefore, the genetic safety of each newly developed formulation should be experimentally verified rather than inferred solely from the properties of its individual components. This approach is consistent with current regulatory frameworks for genotoxicity assessment and internationally accepted guidelines governing the conduct of in vitro and in vivo genetic toxicity studies [15,20,31,32].

A key strength of the present study is the use of standardized OECD TG 487 and TG 471 assays within a unified and harmonized experimental design, ensuring compliance with international regulatory requirements and enabling comparability of genotoxicological data [15,16]. In contrast to previously published studies, which primarily focused on antimicrobial activity or cytotoxicity, the present work provides a comprehensive assessment of both chromosomal and gene-level genetic stability of the D/PVA/I-1 system [13,14].

The present study has several limitations that should be acknowledged. First, the genotoxicity assessment was limited to in vitro assays and did not include in vivo genotoxicity testing. Second, oxidative DNA damage endpoints, such as the comet assay, were not evaluated and could provide additional information regarding DNA integrity. Third, the exposure duration was limited to the timeframes recommended by the OECD test guidelines and therefore may not fully reflect the effects of prolonged exposure. Fourth, although the in vitro micronucleus assay is a well-established method for detecting clastogenic and aneugenic effects, it does not detect gene (point) mutations. Therefore, a comprehensive evaluation of genetic safety requires the use of complementary genotoxicity assays, as implemented in the present study through the combined application of the micronucleus assay (OECD TG 487) and the bacterial reverse mutation assay (OECD TG 471). Finally, although the selected in vitro models provide valuable information on genetic safety, they cannot fully reproduce the complexity of clinical exposure conditions. Future studies should therefore include extended in vivo genotoxicity assessments, as well as a broader toxicological evaluation encompassing oxidative stress analysis, inflammatory responses, tissue-specific effects following long-term exposure, and complementary mechanistic assays to further confirm the safety profile of the D/PVA/I-1 complex.

## 5. Conclusions

The D/PVA/I-1 complex did not induce micronucleus formation in the in vitro micronucleus assay, either in the presence or absence of metabolic activation. Similarly, the bacterial reverse mutation assay (Ames test) yielded negative results for all tested bacterial strains under both metabolically activated and non-activated conditions. It should be noted that the two highest concentrations (5.0 and 2.5 mg/plate) produced pronounced cytotoxic effects, completely suppressing bacterial background growth and revertant colony formation, thereby limiting the interpretation of the assay at these concentrations. Overall, no biologically relevant increase in revertant colony frequency was observed in any of the tested strains compared with the negative control, and no detectable genotoxic or mutagenic effects of the D/PVA/I-1 complex were identified in the performed in vitro test systems. These findings support the further preclinical evaluation of the D/PVA/I-1 complex for biomedical applications. However, additional in vivo genotoxicity studies and complementary mechanistic investigations are required to further confirm its genetic safety. Future studies should also include long-term toxicological evaluations and assessment of the performance of the D/PVA/I-1 complex in biomedical applications, including wound dressings and localized drug delivery systems, to facilitate its further preclinical and clinical development.

## Figures and Tables

**Table 1:** Test strains and their genetic characterization.

Name/Description	Characteristic	Source	Article-No.
*Salmonella typhimurium* TA1535	His G46, rfa, *uvr*B, base-pair substitution strain	ATCC	29629
*Salmonella typhimurium* TA1537	His C3076, rfa, *uvr*B, frameshift mutation strain	ATCC	29630
*Salmonella typhimurium* TA98	His D3052, rfa, *uvr*B, pKM101, frameshift mutation strain	ATCC	14193
*Salmonella typhimurium* TA100	His G46, rfa, *uvr*B, pKM101, base-pair substitution strain	ATCC	14194
*Escherichia coli* WP2 *uvr*A	trp-mutation, DNA repair-deficient strain	ATCC	49979

Abbreviations: His G46, His C3076, His D3052—mutations leading to disruption of histidine biosynthesis (his−); rfa—mutation increasing cell wall permeability; *uvr*B, *uvr*A—defects in DNA repair system; pKM-101—plasmid enhancing mutagenesis level; frameshift mutation strain—strain carrying a frameshift mutation; base-pair substitution strain—strain carrying a base-pair substitution; trp-—defect in tryptophan biosynthesis.

**Table 2:** Positive control substances used in the Ames test.

Chemical and CAS No.	Manufacturer	Lot No.	Purity
Benzo [a]pyrene (CAS No. 50-32-8)	Sigma-Aldrich	SLBP3558V	≥96%
2-Aminoanthracene (CAS No. 613-13-8)	Sigma-Aldrich	STBD3302V	96%
Sodium azide (CAS No. 26628-22-8)	Sigma-Aldrich	BCBQ6198V	≥99.5%
9-Fluorenone (CAS No. 486-25-9)	Sigma-Aldrich	STBC8059V	98%
Methyl methanesulfonate (CAS No. 66-27-3)	Sigma-Aldrich	MKBL6789V	99%

Abbreviations: 2-AA—2-aminoanthracene; B(a)P—benzo[a]pyrene; SA—Sodium azide; 9-FN—9-fluorenone; MMS—methyl methanesulfonate.

**Table 3:** Range of tested concentrations.

Concentration of the D/PVA/I-1 Complex (mg/mL)	S9 (+)	S9 (−)
2.500	4 h exposure + 20 h recovery	4 h exposure + 20 h recovery
1.250	4 h exposure + 20 h recovery	4 h exposure + 20 h recovery
0.625	4 h exposure + 20 h recovery	4 h exposure + 20 h recovery
0.312	4 h exposure + 20 h recovery	4 h exposure + 20 h recovery
0.156	4 h exposure + 20 h recovery	4 h exposure + 20 h recovery
0.078	4 h exposure + 20 h recovery	4 h exposure + 20 h recovery
0.039	4 h exposure + 20 h recovery	4 h exposure + 20 h recovery

**Table 4:** Cytotoxicity of the D/PVA/I-1 complex toward L5178Y cells in the absence and presence of metabolic activation (S9).

Metabolic Activation System	Test Substance	Concentration (mg/mL)	RICC (%)
S9 (−)	Negative control	0	100.0
	Mitomycin C	0.0001	83.3
	D/PVA/I-1	0.039	150.0
		0.078	116.7
		0.156	133.3
		0.312	116.7
		0.625	Not analysable due to excessive cytotoxicity
		1.250	Not analysable due to excessive cytotoxicity
		2.500	Not analysable due to excessive cytotoxicity
S9 (+)	Negative control	0	100.0
	Cyclophosphamide	0.01	100.0
	D/PVA/I-1	0.039	183.3
		0.078	150.0
		0.156	150.0
		0.312	133.3
		0.625	66.7
		1.250	33.3
		2.500	Not analysable due to excessive cytotoxicity

Note: RICC, Relative Increase in Cell Count. RICC was calculated according to OECD TG 487 using viable cell counts determined by the trypan blue exclusion assay. RICC values greater than 100% indicate cell proliferation exceeding that of the concurrent negative control and therefore reflect the absence of cytotoxicity. Concentrations yielding negative RICC values were considered excessively cytotoxic and were not analysable according to OECD TG 487; these are indicated accordingly in the table.

**Table 5:** Results of the micronucleus assay of the D/PVA/I-1 complex in L5178Y cells in the absence and presence of metabolic activation (S9).

Metabolic Activation System	Test Substance	Concentration (mg/mL)	Micronucleated Cells per 1000 Cells		Mean ± SD
			Replicate 1	Replicate 2	
S9 (−)	Negative control	0	5	6	5.5 ± 0.7
	Mitomycin C	0.0001	16	18	17.0 ± 1.4 *
	D/PVA/I-1	0.039	6	6	6.0 ± 0.0
		0.078	5	6	5.5 ± 0.7
		0.156	5	3	4.0 ± 1.4
		0.312	5	6	5.5 ± 0.7
S9 (+)	Negative control	0	4	5	4.5 ± 0.7
	Cyclophosphamide	0.01	16	14	15.0 ± 1.4 *
	D/PVA/I-1	0.039	6	5	5.5 ± 0.7
		0.078	4	4	4.0 ± 0.0
		0.156	2	5	3.5 ± 2.1
		0.312	3	4	3.5 ± 0.7
		0.625	5	4	4.5 ± 0.7

* Statistically significant differences compared with the negative control, *p* < 0.05.

## Data Availability

The original contributions presented in this study are included in the article. Further inquiries can be directed to the corresponding authors.

## Abbreviations

The following abbreviations are used in this manuscript:

2-AA	2-aminoanthracene
9-FN	9-fluorenone
ATCC	American Type Culture Collection
B(a)P	Benzo[a]pyrene
D/PVA/I	Dextrin/polyvinyl alcohol/iodine
DMSO	Dimethyl sulfoxide
FBS	Fetal bovine serum
KCl	Potassium chloride
LB	Luria–Bertani medium
MMS	Methyl methanesulfonate
NADP	Nicotinamide adenine dinucleotide phosphate
NC	Negative control
NBRC	NITE Biological Resource Center
NITE	National Institute of Technology and Evaluation
OECD	Organisation for Economic Co-operation and Development
PBS	Phosphate-Buffered Saline
PC	Positive control
PVA	Polyvinyl alcohol
RICC	Relative Increase in Cell Count
SA	Sodium azide
SD	Standard deviation
TG	Test guideline
